# Microbial community diversity of an integrated constructed wetland used for treatment of sewage

**DOI:** 10.3389/fmicb.2024.1355718

**Published:** 2024-03-18

**Authors:** Nidhi Seth, Sharad Vats, Suman Lakhanpaul, Yasir Arafat, Sudeshna Mazumdar-Leighton, Mansi Bansal, C. R. Babu

**Affiliations:** ^1^Department of Computer Science, Banasthali Vidyapith, Vanasthali, India; ^2^CEMDE, University of Delhi, New Delhi, India; ^3^Department of Biotechnology, Banasthali Vidyapith, Vanasthali, India; ^4^Department of Botany, University of Delhi, New Delhi, India

**Keywords:** sludge, sediment, integrated constructed wetland, sewage, microbial community composition and structure

## Abstract

The microbial community diversity in Constructed Wetland System (CWS) plays a key role in the removal of pollutants from waste water. An integrated functional CWS developed at Neela Hauz Biodiversity Park, Delhi was selected to assess the diversity in composition and structure of microbial community diversity of sludge and sediment of CWS, based on metagenomic approach using 16S rRNA genes. The sediment showed higher diversity than sludge and both formed distinct clusters. The taxonomic structure of the microbial community of CWS is represented by 6,731 OTUs distributed among 2 kingdoms, 103 phyla, 227 classes, 337 orders, 320 families, 295 identified genera, and 84 identified species. The relative abundance of top 5 dominant phyla of sludge and sediment varied from 3.77% (*Acidobacteria*) to 35.33% (*Proteobacteria*) and 4.07% (*Firmicutes*) *to* 28.20% (*Proteobacteria*), respectively. The range of variation in relative abundance of top 5 dominant genera of sludge and sediment was 2.58% (*Hyphomicrobium*) to 6.61% (*Planctomyces*) and 2.47% (*Clostridium*) to 4.22% (*Syntrophobacter*), respectively. The rich microbial diversity of CWS makes it perform better in pollutants removal (59.91–95.76%) than other CWs. Based on the abundance values of taxa, the taxa are grouped under four frequency distribution classes—abundant (>20), common (10–19), rare (5–9), and very rare (1–4). The unique structure of microbial communities of integrated CWS is that the number of abundant taxa decreases in descending order of taxonomic hierarchy, while the number of rare and very rare taxa increases. For example, the number of abundant phyla was 14 and 21 in sludge and sediment, respectively and both communities have only 3 abundant genera each. This is in contrast to 4 and 17 very rare phyla in sludge and sediment, respectively and both the communities have 114 and 91 very rare genera, respectively. The outcomes of the study is that the integrated CWS has much higher microbial community diversity than the diversity reported for other CWs, and the rich diversity can be used for optimizing the performance efficiency of CWS in the removal of pollutants from waste water. Such structural diversity might be an adaptation to heterogeneous environment of CWS.

## Introduction

Globally, the increasing volume of sewage is a major issue that requires sustainable solutions. According to UNDP, 80% of the wastewater enters water bodies and lacks adequate treatment. Improving water quality is one of the many Sustainable Development Goals targeted by UNDP (United Nations Sustainable Development Goal, 2020). It includes lowering the water pollution levels, eliminating dumping, reducing the release of hazardous materials and chemicals, halving the amount of untreated wastewater, and significantly increasing recycling and safe reuse (United Nations Sustainable Development Goal, 2020). Natural wetlands are known for their role in water purification. Consequently, these can be used for sustainable treatment of domestic sewage. In fact, stabilization ponds have been used for treatment of waste water since early 20th century (Vymazal, 2011). The microbial community and the green plants are critical for water purification and sewage treatment in natural wetlands and stabilization ponds, as these are involved in removal of pollutants. In other words, Constructed Wetlands (CWs) are passive biologically engineered systems designed based on the principles of natural wetlands for treatment of waste water. CWs have been widely used since 1960s, as they are simple to operate, easy to maintain, in-expensive and environment friendly (Zheng et al., 2021).

A typical constructed wetland consists of plants, substrates (media), soil, microbes and water. The CWs purify water through physical, chemical and biological processes resulting from interactions among plants, substrates and microbes (Weber, 2016). Based on the type of aquatic plants (vegetation) grown, CWs can be classified into emergent, submerged, floating-leaved, and free floating types (Vymazal, 2010). They are also classified, based on water flow regime and direction into (i) free water surface flow (FWSF or SF) CWs and (ii) subsurface flow (SSF) CWs. The SSF CWs types are further classified into vertical flow (VF) and horizontal flow (HF) types (Vymazal, 2010; Rahman et al., 2020). This classification of CWs has been widely used. There are different designs of CWs but four main configurations have been used in the treatment of waste water across the world. These are FWSF or SF CWs, horizontal subsurface flow CWs (HSSF or HF type), vertical subsurface flow CWs (VSSF or VF type) and hybrid systems involving any combinations thereof such as HF-VF, FWS-HF, and FWS-VF (Weber and Gagnon, 2014; Weber, 2016; Rahman et al., 2020). Besides these four major types, other types like baffled subsurface flow CWs, Aerator CWs, multitrophic free flow engineered wetland and French vertical flow. CWs are also used.

The CWS selected in the present study, basically, belongs to free water surface flow type, different from all the types of CWs in use, with respect to the integration of stabilization ponds and filtration unit with rock filters with CW which has also different configuration. Details of CWS developed and selected are given in materials and methods section.

The CWs are known to remove a wide range of environmental pollutants including emergent pollutants, antibiotics and heavy metals, besides COD, and nutrients like Nitrogen and Phosphorus (Wang et al., 2022). CWs, are, therefore, used as primary/secondary/tertiary treatment of domestic sewage, industrial waste water, mine drainage; land filled leachates, polluted lake water, and river water, dairy waste water, winery effluents and other waste water (Zhao et al., 2020; Parde et al., 2021; Wang et al., 2022).

Microbes are known to be critical in CWs for removal of a variety of pollutants through degradation, assimilation for their growth and multiplication, biosorption, bioaccumulation and speciation transformation and promoting tolerance among plants that remove pollutants in CWs. Microbial communities are distributed throughout CW but there are three main areas of their occurrence within CWs. These are (i) attached, within proximately to or associated with roots of plants (rhizospheric), (ii) within biofilms surrounding the general media and (iii) in the free water or interstitial water. There are diverse environments within CWs that influence functional activities of inherent microbial communities (Faulwetter et al., 2009).

A number of studies were carried out on the assessment of microbial communities of CWs and these were reviewed by Weber (2016). The reviews on the contaminant removal process in SSF CWs by Garcia et al. (2010), microbiology in treatment wetlands by Weber and Gagnon (2014) and microbial biomass, activity and community composition in CWs by Truu et al. (2015) provide the enumeration and function of microbial population in CWs. Recently, Wang et al. (2022) also reviewed published literature on the removal of Nitrogen, Phosphorus, heavy metals and antibiotics and emergent pollutants by microbes, and the processes and taxa (phyla and genera) involved in the removal of pollutants in CWs and also found that phyla such as *Proteobacteria, Bacteroidetes, Actinobacteria*, and *Firmicutes* are dominant groups that contribute to pollution control, and identified pollutants that impact on the diversity of microbial communities in CWs. For example, with respect to removal of antibiotics in CWs, the genera like *Sphingomonas, Sphingorhabdus, Reyranella, Ochrobactrum, Sphingobium, Hyphomicrobium*, and others of *Proteobacteria* were identified (Syranidou et al., 2018; Man et al., 2020; Sauvetre et al., 2020; Zheng et al., 2021). The genera of other phyla such as *Actinobacteria, Firmicutes, Bacteroidetes* were also involved in the removal of antibiotics in CWs (Alexandrino et al., 2017; Syranidou et al., 2018; Santos et al., 2019; Chen et al., 2020; Sauvetre et al., 2020; Wang et al., 2022).

Similarly, the phyla and genera involved in removal of heavy metals, Phosphorus and Nitrogen were also identified (Huang et al., 2017; Lu et al., 2017; Urakawa et al., 2017; Liu et al., 2020; Guo et al., 2021; Wang et al., 2022). For example: The phyla like *Proteobacteria Firmicutes, Actinobacteria, Bacteroidetes* and genera such as *Desulfovibrio*, *Desulfosporosinus*, *Pseudomonas*, *Bacillus*, and others are involved in removal of heavy metals in CWs (Chen et al., 2016; Urakawa et al., 2017; Syranidou et al., 2018; Guo et al., 2021). Taxa such as *Bacteroidetes, Actinobacteria, Proteobacteria, Planctomycetes* are involved in removal of nitrogen from CWs (Zhao et al., 2016, 2021; Huang et al., 2017); whereas phyla like *Proteobacteria*, *Chloroflexi, Actinobacteria*, *Firmicutes*, and genera like *Pseudomonas*, *Gemmatimonas*, *Variovorax* are identified in the removal of phosphorus (Wu et al., 2015; Huang et al., 2017; Lu et al., 2017). Kumar et al. (2023) isolated 32 bacterial isolates belonging to *Bacillus* and one each of *Ralstonia, Citrobacter freundii, Aeromonas veronii, Enterobacter cloacae, Burkholderia cepacia*, and *Priestia flexa* from sediments of CWs.

Weber (2016) reviewed the different methodologies used for assessment of microbial communities in CWs, particularly with respect to enumeration, structure, function and activity. Metagenomic studies of microbial communities of sludge from STPs, and rhizospheric sediment from stabilization ponds and CWs, using 16S rRNA gene sequencing have been carried out (Gao et al., 2016; Yu et al., 2021). However, studies on microbial community diversity of the sludge from the stabilization pond and rhizospheric sediments of CW of the integrated CWS used for *in situ* bioremediation of sewage have not been done. The present paper provides an insight on microbial community diversity in composition and structure of the integrated constructed wetland system used for *in situ* bioremediation of sewage, using metagenomic analysis of 16S rRNA gene.

## Materials and methods

### Integrated CW developed and its location, and collection of samples

#### Constructed wetland system

**Figure 1:**
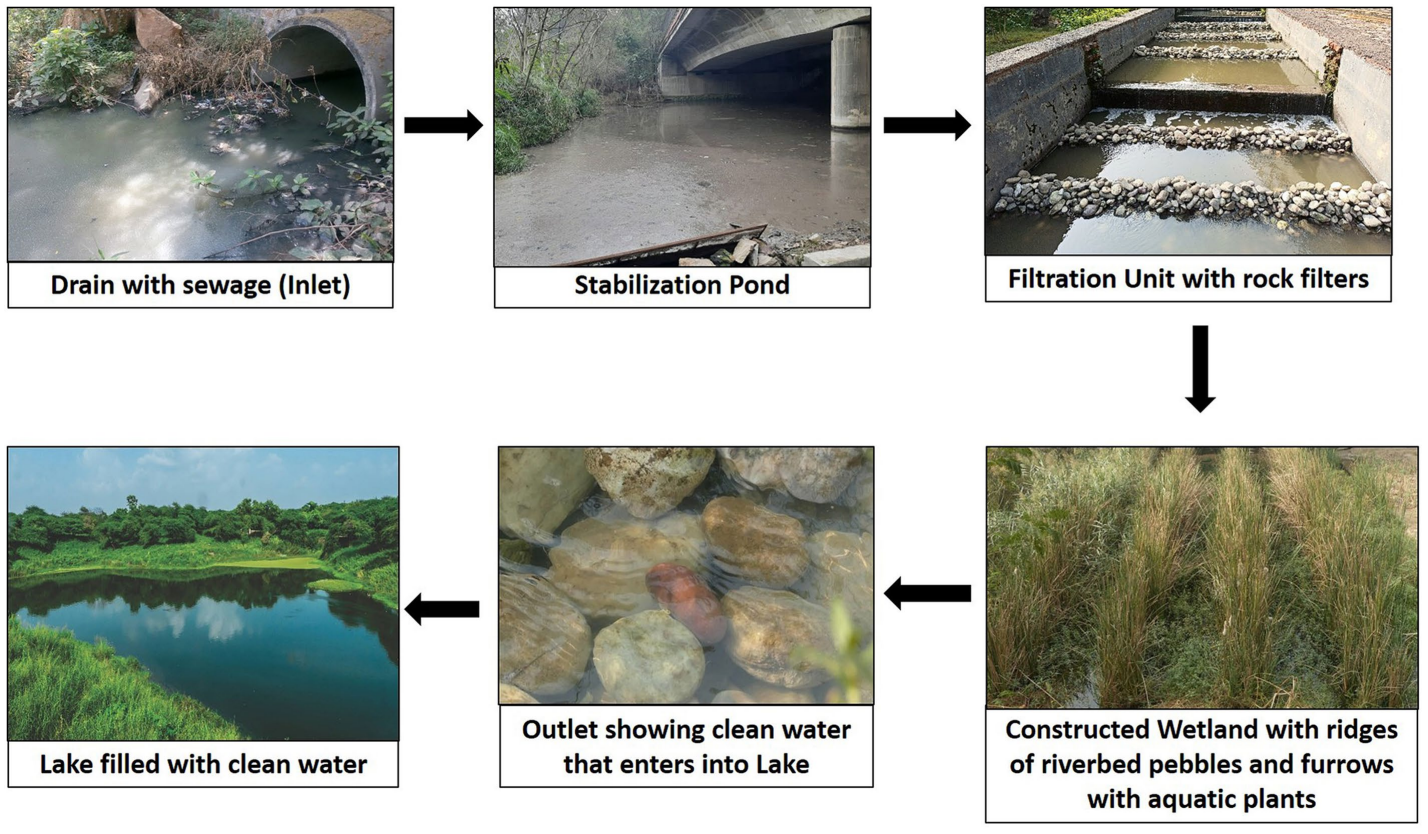
Different components of the integrated CWs selected in the present study.

The CWS selected for sampling of sludge and sediment is of free water surface flow type integrated with stabilization ponds and filtration unit, developed for *in situ* remediation of 1 MLD (million liter per day) sewage. It has a novel design and consists of two stabilization ponds, one filtration unit with rough rock filters and constructed wetland with ridges made up of river bed pebbles and furrows having rooted emergent macrophytes like *Typha latifolia, Typha angustifolia, Phragmites australis, Cyperus papyrus, Scirpus* sp. and rooted floating plants like *Ipomoea aquatica, Alternanthera philoxeroides*, and few floating plants like *Pistia stratiotes* and *Lemna*. The different components of CWS selected in the present study are illustrated in Figure 1.

The removal efficiencies of pollutants by CWS were calculated by

$\text{Removal Efficiency}\;\left(\%\right)=\left[\left(C_{\text{in}}-C_{\text{out}}\right)/C_{\text{in}}\right]\times 100$,

where, *C*_in_ = Concentration of the parameter in inlet and.

*C*_out_ = Concentration of parameter in outlet.

The removal efficiencies of CWS for different water quality parameters like TSS, TDS, COD, BOD, NH_3_-N, and PO_4_-P were 89.57, 72.09, 95.76, 81.15, 79.48, and 59.91%, respectively. The pH was 7.04 in stabilization pond, it was 6.90 in filtration unit and it was 6.72 in the constructed wetland.

The integrated CWS developed is different from the CWs in use by having two stabilization ponds, a filtration unit with rock filters and a constructed wetland with ridges made up of river bed pebbles and furrows with nearly 15 species of aquatic plants and also in its use for *in situ* bioremediation of waste water. It also differs from other CWs in having higher removal efficiency of pollutants within 14 h HRT.

#### Location of CW system

**Figure 2:**
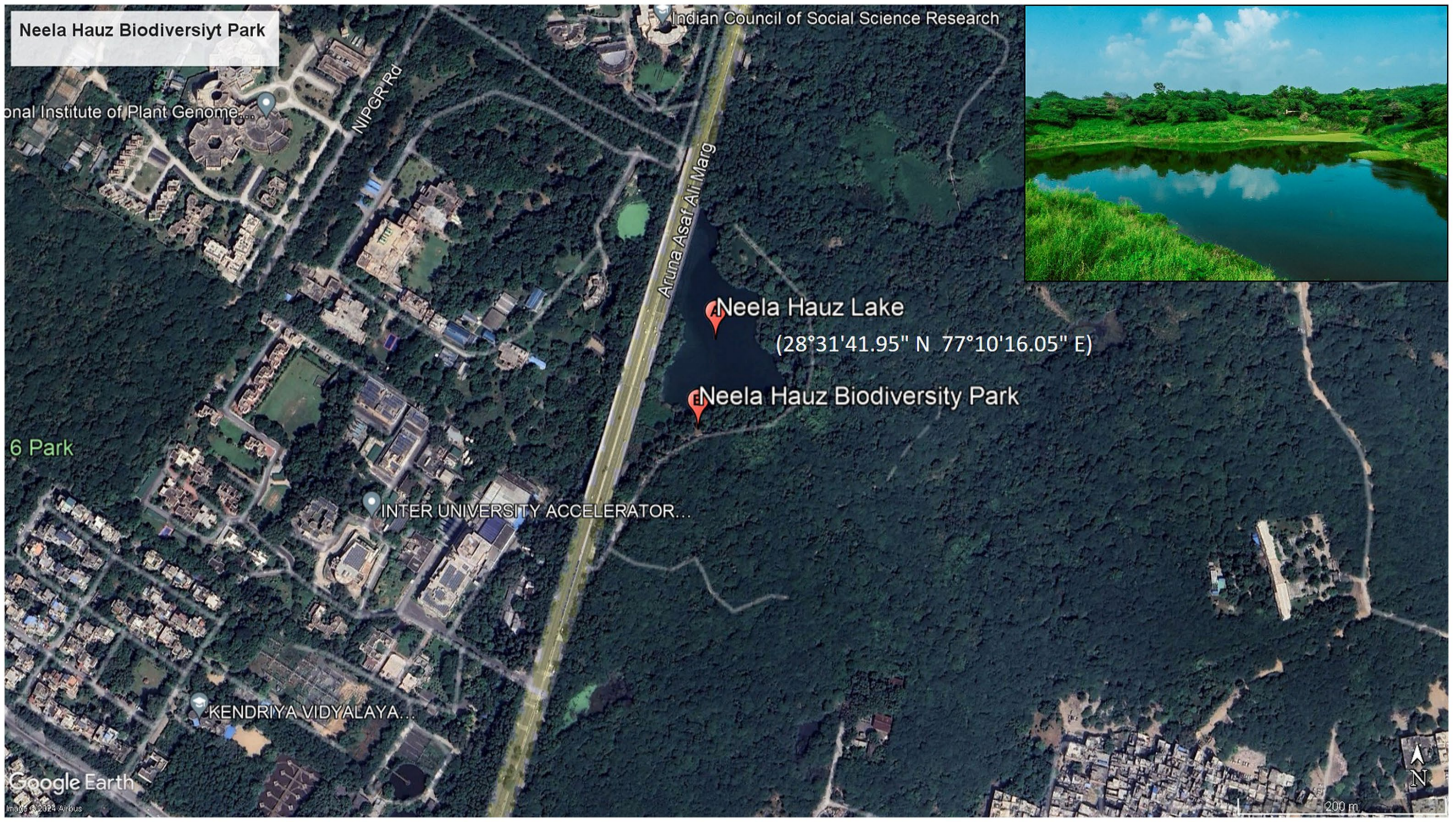
Google earth map of Delhi showing location of Neela Hauz Biodiversity Park with lake (insert).

The integrated CWS is located at Neela Hauz Biodiversity Park, Delhi (Figure 2), and it is an *in situ* bioremediation model. Delhi is located between 28°31′41.95″N and 77°10′16.05″E and has semi-arid climate with average temperature of 38°C and 14°C in summer and winters, respectively; the average relative humidity is 67%, with maximum 82% (in monsoon) and minimum 42% (in summer); the average annual rainfall is 790 mm (31.1 in); the soil is mostly alluvial in nature and the vegetation is mostly of tropical dry deciduous forest to scrub type.

#### Collection of samples

The floating sludge from the stabilization pond 2 of the CW system was collected through vacuum pump and was deposited in a dump. A sample of 1 kg of sludge was transferred from sludge dump into auto-claved (sterilized) plastic bags with help of sterilized large spatula. The rhizospheric sediment was scraped from pulled out plants of *Typha latifolia* and *Phragmites karka* from the constructed wetland and transferred to sterilized plastic bags. Three samples of sludge (N1, N2, and N3) and three samples of sediment (N4, N5, and N6) were collected. These 6 samples collected were stored at −20°C until the extraction of genomic DNA.

### Extraction of genomic DNA and its quantity and quality

QiagenAll Prep Power Viral DNA/RNA kit (Catalog # ID: 28000-50) was used to extract DNA from 250 mg of each of 6 samples collected in RNase free water using the kit protocol.

The quality of isolated DNA was tested by agarose gel electrophoresis using 0.8% agarose gel and also by Nanodrop spectrophotometry. The concentration of DNA was estimated using a DNA IIS Assay Kit on a Qubit 4.0 Fluorometer.

### PCR amplification of 16SrRNA gene and sequencing of amplicons

The isolated DNA together with 3 samples of sludge and 3 samples of sediment were transported to Bionivid (IT Company in Bengaluru, India) for PCR amplification and sequencing. The primer pairs specific to V3 and V4 hyper variable regions of 16S rRNA gene were used.

The set of primers used for PCR amplification were: Forward primer –.

5′-TCGTCGGCAGCGTCAGATGTGTATAAGAGACAGCCTA CGGGNGGCWGCAG-3′.

Reverse primer –.

5′-GTCTCGTGGGCTCGGAGATGTGTATAAGAGACAGGAC TACTVGGGTATCTAATCC-3′.

The PCR amplicon library was constructed with each amplicon of 460 bp. The sequencing of amplicons was done using Illumina MiSeq platform (500X2bp). The data from this study is available at www.ncbi.nlm.nih.gov as PRJNA1052432.

#### Sequence analysis

The generated raw reads were imported into QIIME2 ver 2022.2 (Bolyen et al., 2019). Low-quality reads and chimera sequences were removed using DADA2 plugin in the pipeline. The filtered reads were then clustered into operational taxonomic units (OTUs) with 97% similarity threshold. QIIME2 plugin q2-feature-classifier plugin was used to assign taxonomy to these OTUs. BLAST searches against Greengenes database (ver 13.8) were done to assign taxonomic classification to the OTUs. All the bioinformatic analyses were done using a web-based platform in Microbiome Analyst (Chong et al., 2020).

#### Statistical analysis

Further, the OTU feature tables were used to analyze the microbial community structure in the same pipeline. The α-diversity was estimated for both the samples using Chao I, Shannon and Simpson diversity indices. Rarefactions curves were plotted to assess if the sequencing captured the diversity found in the samples. The microbial communities in samples were clustered using Bray-Curtis distance coefficient to find out the divergence of microbial community structure between sludge and sediment. The relative abundance of different taxa was estimated using the total number of reads in each sample. The top 5 dominant taxa out of all the taxa identified at each taxonomic level, were highlighted. To provide an insight on the structure, the distribution of number of taxa among 4 abundance categories, abundant (>20), common (10–19), rare (5–9), and very rare (1–4) based on abundance values for each taxonomic category was also assessed.

## Results and discussion

### Metagenomics of sludge and sediments of the integrated functional constructed wetland

The composition, structure and function of microbial communities from diverse environmental samples have been an emerging area of research due to its application in addressing the problem of environmental pollution. The metagenomic studies of samples from sludge and sediments of the integrated constructed wetland used for *in situ* bioremediation of sewage were carried out for the first time to assess the similarities and differences in the composition and structure of microbial communities between the sludge from stabilization pond and sediment from CW to formulate consortia of microbial community for the optimization of the pollutant removal by CW. The salient observations on metagenomic analyses of sludge and sediment samples and discussion of these observations in the light of relevant literature are presented below.

### Quality and quantity of genomic DNA extracted from sludge and sediment

The A260/280 ratios for both the samples were between 1.8 and 2.0. The DNA extracted from the samples of sludge and sediment showed discrete bands, when electrophoresed on 0.8% agarose gel. The concentration and yield of DNA among sludge samples ranged from 506 ng/μl to 650 ng/μl and 20,240 ng to 26,000 ng, respectively, while for samples of sediment it varied from 149 ng/μl to 304 ng/μl and 5,960 ng to 12,160 ng, respectively (Table 1). These observations suggest that high quality DNA was extracted and it was suitable for PCR amplification and also adequate for metagenomic study.

**Table 1:** DNA concentration and yield from the sludge and sediment (estimated using Qubit 4.0 Fluorometer).

Sample	Sample codes	Conc. (ng/μL)	Total amount of DNA Available (ng)
Sludge	N1	594	23,760
Sludge	N2	650	26,000
Sludge	N3	506	20,240
Rhizospheric sediment	N4	272	10,880
Rhizospheric sediment	N5	304	12,160
Rhizospheric sediment	N6	149	5,960

### Quality of 16S rRNA gene sequences (reads) generated and OTUs formed

The range of variation in filtered and denoised reads for sludge was 127,163 to 149,203 and 117,251 to 132,136, respectively; for sediment it was 138,737 to 151,946 and 120,592 to 132,107, respectively (Table 2). About 1,174,009 sequences were generated, of which 851,109 sequences were filtered indicating that the sequencing of 16S rRNA gene amplicons generated adequate number of sequences (reads) of good quality. The mean length of reads was 454.76 bp with minimum and maximum length of 275 and 413 bp, respectively. These sequences were clustered into 6,731 OTUs (97% similarity).

**Table 2:** Filtered and denoised reads.

Sample	Input	Filtered reads	Percentage reads of input passed filter	Denoised	Merged	Percentage of input merged	non-chimeric	Percentage of input non-chimeric
Sludge	181,799	133,871	73.64	117,251	59,782	32.88	26,110	14.36
Sludge	181,712	127,163	69.98	111,994	55,833	30.73	25,624	14.1
Sludge	199,136	149,203	74.93	132,136	72,959	36.64	32,422	16.28
Sediment	207,594	150,189	72.35	131,085	70,203	33.82	34,152	16.45
Sediment	214,220	151,946	70.93	132,107	70,658	32.98	33,114	15.46
Sediment	189,548	138,737	73.19	120,592	64,405	33.98	32,421	17.1

**Figure 3:**
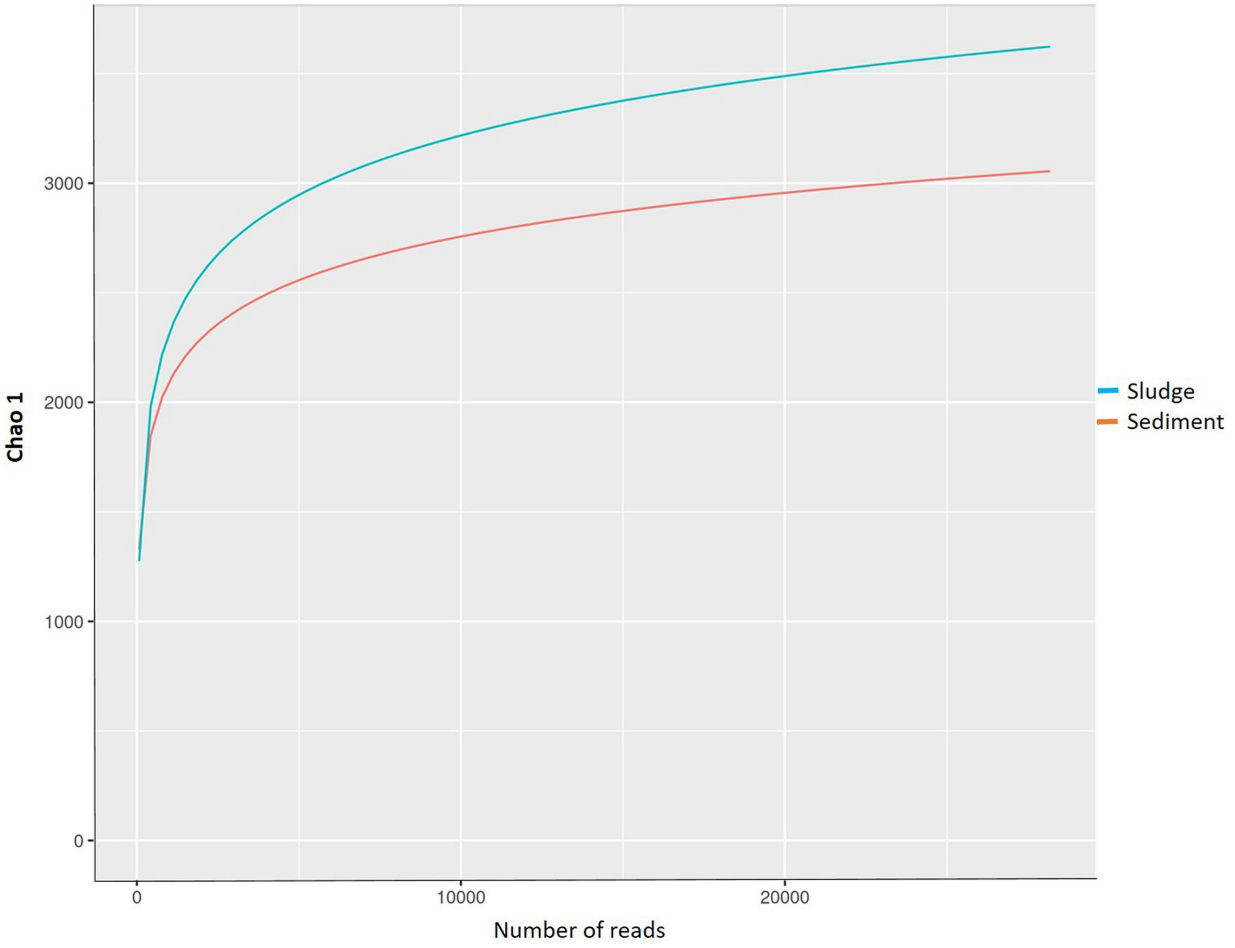
Rarefaction curves for the sludge and sediments.

The rarefaction curves were flattened at 3,000–3,500 OTUs against 20,000 reads, and represented the depth of sequencing (Figure 3) and indicated that the sequences generated captured the diversity found in the samples. Out of the total OTUs 6,731 (species), 3,052 species (41.45%) were recorded for sludge only, whereas 3,679 species (54.65%) were recorded for sediment only, and just 352 (5.4%) species were common to both the sludge and sediment. The sediment showed 9.35 percent more OTUs than sludge. These observations indicate that both sludge and sediment have microbial communities with rich diversity, and the community of sediment showed higher diversity than that of the sludge at OTU level.

Cao et al. (2017), Jeong and Ham (2017), Usharani (2019), and Kumar et al. (2023) carried out similar studies and reported lower concentrations of DNA and higher number of reads from sediments of CWs. However, Usharani (2019) reported lesser number of reads (726838) in CW system treating sewage as compared to the number of reads generated in the present study. Zhao et al. (2017) reported low number of OTUs (2419.33) in rhizospheric sediment of macrophytes and also for bulk sediment (3209.67) than the number of OTUs (6514) recorded for both sludge and sediment communities together of the integrated CWS. The high number of OTUs (3679) in the sediment community might be due to sampling of sediment from rhizosphere of rooted macrophytes of CW.

### Alpha diversity of microbial communities

The species richness in terms of Chao index was higher (3531.71) for sediment than that of sludge (2918.81); similarly, the evenness expressed in terms of Shannon index was also higher (7.70) for sediment than that of sludge (7.52); the diversity expressed in terms of Simpson index (evenness + richness) was also higher (0.9993) in sediment than that of sludge (0.9992). The sediment also showed higher number of OTUs than that of sludge. These results indicate that sediment has more species rich and diversified community than that of sludge because it was sampled from rhizosphere of aquatic plants (macrophytes) that enrich nutrients and oxygen. The α-diversity observed in the present study is more or less similar to that reported by other workers. For instance, Gao et al. (2016) reported Shannon index ranging from 7.32 to 7.99 and the Chao I index ranging from 31,196 to 43,581 and the OTUs ranged from 7,156 to 10,510 for activated sludge from four municipal waste water treatment plants. Shanks et al. (2013) also reported Chao I index values ranging from 2,332 to 3,196 for untreated waste waters collected from different locations in USA. Yu et al. (2021) reported that rhizospheric sediments showed higher number of species 9,560–12,100 as compared to non-rhizospheric sediments; they also reported higher Chao I index (11,600–16,400), Shannon index (11.3–12.0) and Simpson index (0.9971–0.9979) for rhizospheric sediment in contrast to non rhizospheric sediment which showed ChaoI index ranging from 11,300 to 15,900, Shannon index ranging from 11.1 to 12.0 and Simpson index ranged from 0.9970 to 0.9977. Although Chao I index values reported by Yu et al. (2021) are higher than the value obtained in the present study for rhizospheric sediment and sludge, but, the Shannon index value for the integrated CW is higher than the values reported by Yu et al. (2021). These differences and similarities in species indices of microbial communities of samples studied by different workers might be due to different plants used in CWs, quality of sewage, types of pollutants and other environmental conditions prevailing in CWs.

### Taxonomic structure and composition of microbial communities

The metagenomic analysis of 16S rRNA genes of microbial communities in sludge and rhizospheric sediment of CW revealed the taxonomic structure of microbial community present in CWS, and it is represented by 6,731 OTUs (species), 295 identified genera, 320 families, 337 orders, 224 classes, and 103 phyla and 2 kingdoms.

**Figure 4:**
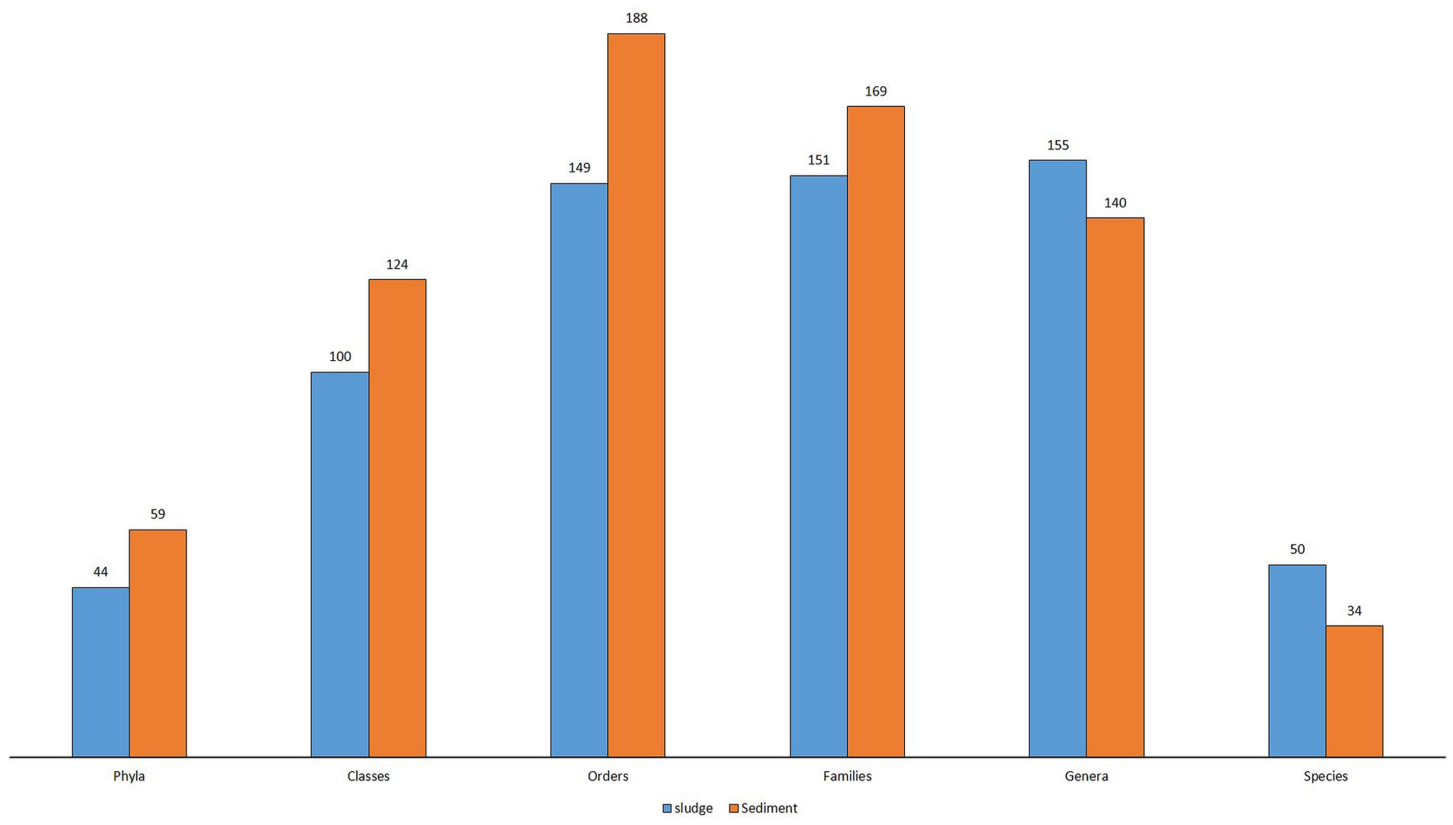
Histogram showing taxonomic structure of microbial communities of sludge and sediment.

Even at the Kingdom level the diversity was higher in the sediment [25 Archaea (0.68%) and 3,654 bacteria (99.32%)] than in the sludge [3 Archaea (0.028%) and 3,049 bacteria (99.9%)]. The abundance of bacteria is several times higher than Archaea. Rana et al. (2023) reported 85% OTUs (4,829) belong to Bacteria in the sediment of *Typha latifolia*. The taxonomic structure of sludge microbial community was represented by 44 phyla, 94 classes, 128 orders, 120 families, 96 identified genera and 21 identified species, whereas the sediment microbial community was represented by 59 phyla, 124 classes, 188 orders, 169 families, 140 identified genera, and 34 identified species (Figure 4). There is a marked difference in the taxonomic structure of microbial community between sludge and sediment and also different from the structure reported by other workers. Gao et al. (2016) reported 33 phyla, 87 orders, 187 families and 627 genera from activated sludge of 4 STPs. In the present study more phyla and orders were reported but the number of genera was less than the number reported by Gao et al. (2016). This is because many of the OTUs were not identified at the genus level. Nascimento et al. (2018) reported 68 phyla, 164 classes and 665 genera from the sewage sludge, but in the present study lesser number of phyla, classes and genera were reported from both sludge and sediment. Zhao et al. (2017) reported the top 30 abundant genera from rhizospheric sediments of submerged macrophytes.

### Microbial community diversity at phylum level

A total of 103 phyla were recorded for both sludge and sediment, of which 44 were found in sludge and 59 (57.28%) were found in sediment. The 42 (40.7%) phyla found in sludge were also found in the sediment, which had 17(16.5%) unique phyla in contrast to 2 (1.94%) unique phyla of sludge community. The range of variation in relative abundance values was 0.03–35.33% and 0.03–28.20% for sludge and sediment, respectively, across the phyla (Supplementary Tables S1, S2).

The rich diversity in rhizospheric sediment microbial community (59 phyla with 16.5% unique phyla) as compared to that of sludge (42 phyla with 1.94% unique taxa) is due to aerobic condition in the root zone and also higher nutrient rich environment, besides diversified ecological niches. In both sludge and sediment, the most dominant phylum was *Proteobacteria* with relative abundance of 28.20% and 35.32% in sludge and sediment communities, respectively.

Zhao et al. (2017) also showed that *Proteobacteria* was the dominant phylum in the microbial community of rhizospheric and bulk sediments and also observed differences in diversity between rhizospheric and bulk sediments. Similar observations have also been made by many workers not only with sediments (Choi et al., 2021) but also with rhizospheric soils of crop plants (Bakker et al., 2015).

**Figure 5:**
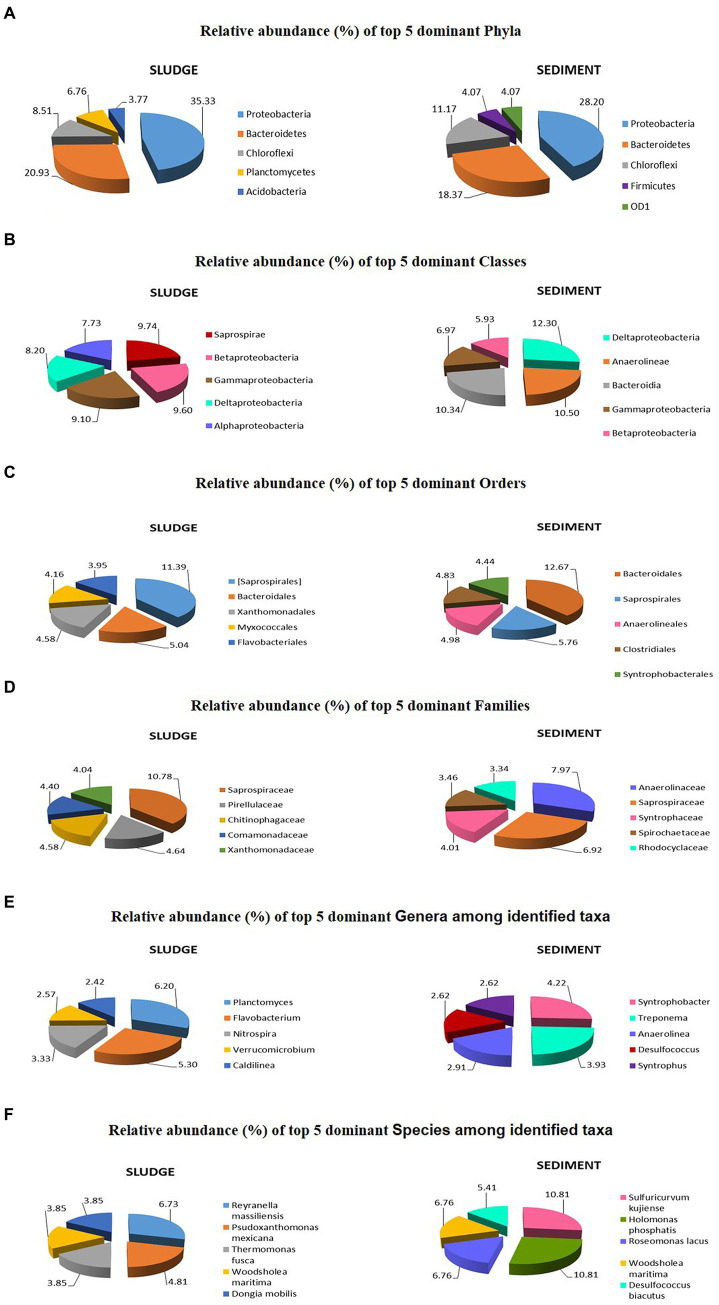
Top 5 dominant taxa and their relative abundances in microbial communities of sludge and sediment: Phylum **(A)**; Classes **(B)**; Orders **(C)**; Families **(D)**; identified Genera **(E)**; and identified Species **(F)**.

The top 5 most dominant phyla in the sludge were *Proteobacteria* (35.33%), *Bacteroidetes* (20.93%), *Chloroflexi* (8.51%), *Planctomycetes* (6.76%), and *Acidobacteria* (3.77%), but in the sediment the top 5 phyla include the first three phyla of sludge with different relative abundance values [*Proteobacteria* (28.20%), *Bacteroidetes* (18.37%), *Chloroflexi* (11.17%)] and the 4th and 5th dominant phyla were *Firmicutes* (4.07%) in place of *Planctomycetes* and ODI (37.7%) instead of *Acidobacteria*, *Verrucomicrobia* (3.92%); and *Planctomycetes* (3.45%) occupy 6th and 7th positions, respectively in the sediment (Supplementary Tables S1, S2). Similar patterns of variation were reported by Yu et al. (2021), who reported that rhizospheric sediment had higher diversity than the non rhizospheric sediments. Rana et al. (2023) also made similar observation relating to the diversity at phylum-level in microbial community of the Rhizospheric sediment of *Typha latifolia*. Many workers (Gao et al., 2016; Liang et al., 2017; Meerbergen et al., 2017; Zhang et al., 2021) found *Proteobacteria, Bacteroidetes*, and *Actinobacteria* were dominant in domestic sludge but *Planctomycetes, Chloroflexi, Acidobacteria*, and *Chlorobi* were predominant in industrial sludge. In the present study, the samples of integrated CW showed all the above phyla of domestic as well as industrial sludge except for *Chlorobi* (Figure 5A). The relative, abundance of different phyla is different between sludge and sediment (Supplementary Tables S1, S2) suggesting different environmental factors that influence microbial diversity within CWs.

The pattern of variation in the structure and composition of microbial community in terms of distribution of phyla among four abundance categories is markedly different between sludge and sediment. For example, 31.81% phyla come under abundant category in the case of sludge, and it was 35.59% phyla for sediment; the common phyla were 18.18% and 20.33% in sludge and sediment, respectively (Table 3).

**Table 3:** Distribution of total number of common and unique taxa at different taxonomic categories among microbial communities of sludge and sediment.

			Sludge		Sediment	
	Total taxa	Percentage of common taxa in sludge and sediment	Total sludge	Percentage of unique taxa	Total sediment	Percentage of unique taxa
Phylum	103	42(40.7%)	44 (42.71%)	2(1.94%)	59(57.28%)	17 (16.5%)
Class	224	94 (41.96%)	100 (44.64%)	6 (2.68%)	124 (55.35%)	30 (13.39%)
Order	337	128 (37.98%)	149 (44.21%)	21 (6.23%)	188 (55.78%)	60 (17.80%)
Family	320	120 (37.5%)	151 (47.18%)	31 (9.69%)	169 (52.81%)	49 (15.31%)
Genera	295	96 (32.54%)	155 (52.54%)	59 (20.00%)	140 (47.45%)	43 (14.58%)
Species	84	21 (25%)	50 (59.52%)	29 (34.52%)	34 (40.47%)	13 (15.48%)

The top 5 dominant phyla found in the sludge and sediment were also the top 5 phyla under abundant category; in sludge the top 5 common phyla were *Gemmatimonadetes, TM6, Cyanobacteria, Chlamydiae* and *WS3*; while *Nitrospirae, Gemmatimonadetes, Armatimonadetes, Cyanobacteria*, and *Euryarchaeota* were the top 5 common phyla in the sediment; about 18.8% (8) of phyla in the sludge and 20.33% (12) phyla in the sediment were common (Table 4).

**Table 4:** Frequency distributionof Phyla, Classes, Orders, Families, Genera, and Species (expressed as counts and percentages) among 4 frequency classes.

	Phyla Sediment	Phyla Sludge	Class Sediment	Class Sludge	Order Sediment	Order Sludge	Family Sediment	Family Sludge	Genera Sediment	Genera Sludge	Species Sediment	Species Sludge
Total number	59	44	124	100	188	149	169	151	140	155	34	50
Very rare (1–4)	17 (28.81%)	18 (40.9%)	44 (35.48%)	41 (41%)	96 (51.06%)	64 (42.95%)	94 (55.62%)	76 (50.33%)	91 (65%)	114 (73.54%)	30 (88.23%)	48 (96%)
Rare (5–9)	9 (15.25%)	4 (9.09%)	29 (23.38)	20 (20%)	39 (20.74%)	30 (20.13%)	33 (19.52%)	34 (22.51%)	27 (19.28%)	30 (19.35)	4 (11.76%)	2 (4%)
Common (10–19)	12 (20.33%)	8 (18.18%)	23 (18.54%)	14 (14%)	24 (12.76%)	24 (16.10%)	24 (14.20%)	23 (15.23%)	19 (13.57%)	8 (5.16%)	Nil	Nil
Abundant >20	21 (35.59%)	14 (31.81%)	28 (22.58%)	25 (25%)	29 (15.42%)	31 (20.80)	18 (10.65%)	18 (11.92%)	3 (2.14%)	3(1.93%)	Nil	Nil

The percent of rare and very rare, phyla were different between sludge and sediment (Table 4). For example, the rare and very rare phyla were represented by 40.9% and 9.09%, respectively for sludge, and for sediment the rare and very rare taxa were 15.25% and 28.81%, respectively. The rare category included phyla like *Caldithrix, Synergistetes, NKB19* and *GN02*, and very rare category was represented by *Parvarchaeota, Caldiserica, Elusimicrobia*, and *Fibrobacteres* in the sludge community. For sediment community the rare phyla included, *Synergistetes, Tenericutes, Parvarchaeota, GOUTA4* and very rare phyla were represented by *Thermotogae, Fusobacteria, Lentisphaerae, Crenarchaeota* (Table 4; Supplementary Tables S1, S2). These phyla are different from those reported by Zhao et al. (2020), Garbeva et al. (2008), and Zhao et al. (2017) who also categorized the different taxonomic groups under two categories—the general (with more than 11 sequences) and rare (with one sequence only). In their study, they reported that the composition of bacterial community with respect to rare category was different for different samples but with respect to general category it was not similar to that of overall bacterial community; further they found that the rare category taxa from rhizospheric sediments of different submerged vascular plants were clustered together suggesting similarity, but these were different from the overall bacterial community; they concluded that bulk sediment had more diversity than the rhizospheric sediment. In the present study the percentages of abundant and common phyla are more as compared to that of rare and very rare phyla in both sludge and sediment but different in composition between sludge and sediment communities (Table 4).

It is difficult to explain the presence of few abundant phyla, and slightly less than 50 percent rare and very rare taxa. This is perhaps the inherent property of microbial community structure in sludges and sediments of CWs.

The 15 unique phyla, most of which were very rare and rare category, were found only in sediment and these were absent in the sludge. Since the sediments were from the rhizosphere of vegetated CW, it is likely that these unique phyla inhabit rhizospheres of different aquatic plant species. Such unique phyla specific to sludge or sediment were reported by Cao et al. (2017) who recorded that the sediments of 4 wetlands showed rich diversity in the number of phyla. Among the unique phyla of sediment, in the present study, the phylum *TPD 58* was common, *WWE1 and NC10* were rare, and the rest 12 (*AC 1, Crenarchaeota, FCPU426, Hyd24-12, Kazan-3B-28, OP1, OP9, PAUC34f, TA06, Thermotogae, TPD-58, ZB3*) were very rare. These 15 unique phyla might be specialists in function, whereas all others are generalists.

### Microbial community diversity at class level

The total number of classes recorded was 224, of which only 100 classes were found in sludge and the remaining 124 were observed in the sediment. Both the sludge and sediment shared 94 classes in common; sediment showed 30 (13.39%) unique classes, whereas 6 (2.68%) classes were unique for sludge (Table 3).

The top 5 dominant classes in the sludge were *Saprospirae, Betaproteobacteria, Gammaproteobacteria, Deltaproteobacteria*, and *Alphaproteobacteria* with relative abundance values ranging from 7.73 to 9.74%.

In the sediment, the top 5 dominant classes were *Deltaproteobacteria, Anaerolineae, Bacteroidia, Gammaproteobacteria*, and *Betaproteobacteria* with relative abundance values ranging from 5.93 to 12.30% (Supplementary Tables S1, S2). Jeong and Ham (2017) reported *Anaerolineae* and *Dehalococcoidetes* of *Chloroflexi* phylum in CW adjacent to polluted nursery, but in the present study *Anaerolineae* was reported as second dominant class in the sediment. They also reported all the four classes of *Proteobacteria* in both CWs but in the present studies four classes of *Proteobacteria* were found in sediment but *Alphaproteobacteria* was not in the top 5 dominant classes (Figure 5B). Further the relative abundances of different classes are different between sludge and sediments (Supplementary Tables S1, S2). The range of variation in relative abundance of classes found in sludge and sediment were 0.03 to 9.74% and 0.03 to 12.30%, respectively, across the classes.

Most of the unique classes fall under rare and very rare categories suggesting that they may represent specialists. The abundant and common classes are same in both the sludge and sediment communities suggesting that these are generalists. Further, both sludge and sediment showed higher number of rare and very rare classes as compared to abundant and common classes (Table 4). In other words, the structure of microbial community showed lesser number of abundant classes and higher number of rare and very rare classes. The percent of classes belonging to abundant and common categories were higher (Table 4) for sludge as compared to that of sediment. The percent of rare and very rare classes for sludge was 75% in contrast to 77.4% observed in sediment.

These qualitative and quantitative differences at class level between sludge and sediment suggest that different environmental factors operate in different components of integrated CW that influence the composition and structure of microbial communities. It has been reported that pH influences the microbial community structure and composition (Fierer and Jackson, 2006). At higher pH value (by liming)–, an important method for sludge hygienization or pathogen control—the diversity decreases (Farzadkia and Bazrafshan, 2014). Many workers also showed that pH affected the diversity and structure in sewage sludge (Maspolim et al., 2015). Infact, Lauber et al. (2009) observed that relative abundance of *Alpha, Beta*, and *Gamma Proteobacteria* classes and phyla *Acidobacteria, Actinobacteria*, and *Bacteroidetes* depends upon pH but not on the source location of the sewage sludge. It is likely that pH may influence the nutrient availability and enzymatic processes that are involved in metabolism of microbes resulting in modulation of microbial community composition and structure (Madigan et al., 2016). The patterns of variation in the microbial community diversity at class level suggests that rhizospheric sediment communities are more diversified than that of the sludge which might be due to differences in environmental factors like pH of water which is different between stabilization pond (7.94%) and CW (6.72%), from which the sludge and sediment were sampled, respectively. The details of physico-chemical properties of sewage in different components of the integrated CWS and the removal efficiencies of pollutants by different components have been given in a paper communicated for publication.

### Microbial community diversity at order level

The total number of orders recorded was 337, of which 128 orders were common to both sludge and sediment. Sediment showed 60 (17.80%) unique orders, whereas 21(6.23%) orders were unique for sludge. As in phyla and classes, the diversity at the order level was also very high (188 orders) in the sediments which constituted 55.78% of the total orders recorded (Table 3) suggesting that rhizospheric sediment microbiome is richer in diversity than non-rhizospheric sludge. Similar observations were made by Yu et al. (2021), who reported that complexity, composition, and structure of microbial community in rhizospheric sediment is higher than non rhizospheric sediments.

The top 5 dominant orders in the sludge were *Saprospirales, Bacteroidales, Xanthomonadales, Myxococcales*, and *Flavobacteriales* with relative abundance ranging from 3.95 to 11.39%, and all of them fall under abundant category. For sediment, the top 5 dominant orders were *Bacteroidales, Saprospirales, Anaerolineales, Clostridiales*, and *Syntrophobacterales* with relative abundance ranging from 4.44 to 12.67% (Figure 5C), and all of them fall under abundant category (Table 4). The number of unique orders, the number of orders in the abundant category and top 5 dominant orders with relative abundance values also suggest that the microbial community in rhizospheric sediment is highly diversified than that of sludge. This can be explained on the basis of diverse ecological niches in rhizosphere which harbor a complex of microbial communities (Bais et al., 2006; Nie et al., 2015; Chen et al., 2016). The range of variation in relative abundance for sludge and sediment communities were 0.04–11.38% and 0.04–12.67%, respectively, across the orders. The different orders and their relative abundance found in sludge and sediment are given in Supplementary Tables S1, S2.

Cao et al. (2017) reported that the main orders found in the river wetlands and constructed wetlands were *Burkholderiales, Rhodocyclales* of *Betaproteobacteria, Desulfobacterales, Desulfuromonadales, Syntrophobacterales, Myxococcales* of *Deltaproteobacteria. Saprospirales, Bacteroidales, Cytophagales, Flavobacteriales*, and *Sphingobacteriales* of *Bacteroidetes*. The other dominant orders found in the wetland studied by them were *Phycisphaerales, Gemmatales, Pirellulales, Planctomycetales* of *Planctomycetes, Acidobacteria-6* of *Acidobacteriales*, *Actinomycetales* of *Actinobacteria, Lactobacillales, Clostridiales* of *Firmicutes* and *Thermodesulfovibrionales* of *Nitrospirae*. In the present study, almost all the mentioned above orders are found and their relative abundance values are higher and fall under abundant and common categories.

Usharani (2019) reported only 19 orders in case of CWs planted with *Cyperus alternifolius* in contrast to 337 orders recorded in the present studies; she also reported *Cytophagales* as predominant order but in the present studies it is also found but it is not the dominant order. It may be noted that other orders mentioned by Usharani (2019) are also found in both sludge and sediments of CWS. Gao et al. (2016) reported 87 orders in activated sludge of STPs, where they found *Sphingobacteriales, Burkholderiales, Bacteroidales, Xanthomonadales, Pseudomonadales, Flavobacteriales, Clostridiales, Rhizobiales, Rhodobacterales, Rhodocyclales*, and *Sphingobacteriales* as the most dominant orders. These orders were also found in sludge and sediment of CWS but the abundance values were markedly different from those reported by Gao et al. (2016) For example, abundance value of *Sphingobacteriales* was 53 (2.22%) in case of sludge and in case of sediment it was 54 (2.09%); for *Rhodocyclales* it was 53 (2.22%) for sludge but it was 25 (0.97%) for sediment. This is in contrast to the values *viz.* 106 (30.2%) and 138 (32.5%), reported by Cao et al. (2017), for *Sphingobacteriales* and *Rhodocyclales*, respectively. The values given in parenthesis represent relative abundance. The other orders reported by Gao et al. (2016) were also found in sludge and sediment of CW.

The numbers of orders that fall under 4 categories of abundance are given in Table 4. The abundant orders constitute 20.80% (31) and 15.42% (29′) for sludge and sediment, respectively; 18.79% orders of sludge and 12.76% of sediment were common; the bulk of orders fall under rare and very rare categories of abundance (Table 4).

The differences in the relative abundance values for abundant and common orders were markedly different between sludge and sediment but for rare and very rare categories, the differences between sludge and sediment communities not markedly different (Table 4).

For example, in sludge the relative abundance of *Saprospirales* was 11.39%, *Bacteroidales* was 5.04% *and Xanthomonadales* was 4.58% in contrast to sediment where the relative abundance of *Bacteroidales* was 12.67%*, Saprospirales* was 5.76% and *Xanthomonadales* was 1.97%. In the rare category *Pseudomonadales* was represented by relative abundance of 3.72% in sediment but its relative abundance was 13.42% in sludge. Similar patterns were observed by Kumar and Chandra (2020) for bacterial communities associated with *Saccharum arundinaceum* grown on organometallic pollutants-rich hazardous distillery sludge. This characteristic community structure is perhaps inherent feature of microbial communities that sustain high diversity under limiting DO and other nutrients. Such diversified communities may be highly efficient in removal of pollutants.

### Microbial community diversity at family level

The total number of families recorded was 320, of which 151 families were observed in sludge and 169 families were found in sediment; of the total 320 families, 120 families were common to both the samples; 49 (15.31%) taxa were restricted to sediment and 31 (9.69%) families were unique to sludge. In other words, 15.31% of families were specific to sediment in contrast 9.69% families that were unique to sludge (Table 3).

As in other higher taxonomic groups, the sediment showed higher number of families (169) than the sludge (151) suggesting higher diversity in rhizospheric sediment than in sludge. This is also evident from higher number of families under abundant and common categories and higher number 49 (15.31%) of unique taxa in sediment as compared to that of sludge 31 (9.69%) (Table 3). Gao et al. (2016) also reported 187 families in activated sludge of STPs from China. Usharani (2019) reported only 13 identified and 6 unidentified families from sediment of sewage. In the present study higher number of families were reported as compared to the number of families reported by other workers.

There is a marked difference in the number of families between sludge and sediment of the integrated CWS which might be due to the fact that sludge was sampled from stabilization pond which did not have vegetation and sediment was sampled from CW having vegetation.

The high number of families reported in the present study is because of combined diversity of two microbial communities—one inhabiting stabilization pond and the other found in vegetated CW—whereas other workers reported diversity of microbial community of either activated sludge or sediment of CW.

The top 5 dominant families with their respective abundance and relative abundance (given in parenthesis) found in sludge and sediment are as follows: The dominant families in sludge community were *Saprospirae* 179 (10.78%), *Xanthomonadaceae* 67 (4.04%), *Comamonadaceae* 73 (4.40%), *Chitinophagaceae* 76 (4.58%), *Pirellulaceae* 77 (4.64%), and *Rhodocyclaceae* 53 (3.19%); the dominant families in sediment community were *Anaerolineaceae* 129 (7.97%), *Saprospirae* 42 (*6.92%*)*, Syntrophaceae* 65 (40.014%), *Spirochaetaceae* 56 (3.46%), *Rhodocyclaceae* 54 (3.34%), and *Desulfobacteraceae* 53 (3.27%) (Figure 5D). Except *Comamonadaceae*, all the dominant families reported for sludge and sediment in the present study are different from dominant families reported by Gao et al. (2016) and Usharani (2019).

The range of variation in relative abundance for sludge and sediment were 0.06–10.78% and 0.061–7.97%, respectively across the families (Supplementary Tables S1, S2).

The abundance values of different families between sludge and sediment were markedly different. For example, *Clostridiaceae* showed relative abundance of 0.78% in sludge but it was 1.42% in sediment; for some families there were no differences in relative abundance values between sludge and sediment (Supplementary Tables S1, S2).

The numbers of families that fall under 4 categories of abundance are given in Table 4. The abundant families constitute 11.92% (18) and 10.65% (18) for sludge and sediment, respectively; 15.23% (23) families of sludge and 14.20% (24) of sediments were common and bulk of families fall under rare and very rare categories of abundance.

### Microbial community diversity at genus level

A total of 295 genera were recorded for both sludge and sediment, and this number was less than the number of orders and families recorded; of the 295 genera, 140 genera were recorded for sediment and 155 were recorded for sludge. About 32.54 percent (46) genera were common to both sludge and sediment. The number of unique genera was 43(14.58%) and 59 (20%) for sediment and sludge, respectively (Table 3). This suggests that many of the unidentified genera (Supplementary Tables S3, S4) are either novel or new taxa. Many of OTUs were not identified at genus level and hence their number is less as compared to number of families.

Some workers (Gao et al., 2016; Nascimento et al., 2018; Yu et al., 2021) reported higher number of genera than families from rhizospheric sediments and sediments from STPs. This is perhaps due to identification of more OTUs up to genus level. It may be noted that out of 3,052 total OTUs in sludge and 3,680 OTUs in sediment, the number of identified OTUs upto species was 50 and 34, respectively. These observations suggest that unidentified OTUs at the species and genus levels are more in sediment than in sludge. The sediment may have more new and novel taxa than the sludge.

With respect to identified genera, the sludge showed higher diversity both in terms of total number and percent of unique taxa than in the sediment (Table 3). This suggests that sludge has greater diversity.

The most dominant genera are different between sludge and sediment. For example: in sludge the top 5 genera were *Planctomyces* (41), *Flavobacterium* (35), *Nitrospira* (22), *Verrucomicrobium* (17), *Caldilinea* (16), and *Hyphomicrobium* (16), while in sediment the top 5 dominant genera were *Syntrophobacter* (29), *Treponema* (27), *Anaerolinea* (20), *Desulfococcus* (18), *Syntrophus* (18), and *Clostridium* (17) (Figure 5E). The abundance values of taxa are given in parenthesis. The range of variation in relative abundance for sludge and sediment were 0.15–6.20% and 0.14–4.22%, respectively, across the genera (Supplementary Tables S1, S2).

Similar observations were made by other workers who reported different dominant genera between rhizospheric and non rhizospheric communities (Gao et al., 2016; Nascimento et al., 2018).

The distribution of number of genera under the 4 categories of abundance, are markedly different within and between sludge and sediment (Table 4). For example, abundant genera were 1.93% (3), common genera were 5.16% (8), the rare genera were 19.35% (30), and very rare genera were 73.54% (114) for sludge; for sediment the number of abundant genera were 2.14% (3), common genera were 13.57% (19), the rare genera were 19.28% (27) and very rare genera were 65% (91).

### Microbial community diversity at species level

The diversity among identified species was higher (59.5) in sludge than in the sediment (40.47%), where only 34 species were identified out of 3,654 OTUs. Therefore, only 1.2% of OTUs were identified upto the specific rank suggesting that the diversity observed in the sludge and sediment was unique and may represent new and novel taxa (Supplementary Tables S3, S4). In fact, the numbers of unidentified OTUs were 3,620 and 2,995 for sediment and sludge, respectively. Both the sludge and sediment shared 21 common species and the number of species unique to sediment was 13 (15.48%) and for sludge it was 29 (34.52%) (Table 3).

It may be noted that most of the workers on metagenomics of sludge/sediments of CWs did not report the species, and identification of OTUs was done from kingdom to genus level only (Muyzer and Stams, 2008; Shanks et al., 2013; Lam et al., 2015; Gao et al., 2016; Yu et al., 2021; Kumar et al., 2023).

However, in some cases, particularly dealing with functions, species were identified (Wang et al., 2022).

The top 5 dominant species in the sludge was *Reyranella massiliensis*, *Psudoxanthomonas mexicana, Thermomonas fusca*, *Woodsholea maritima*, and *Dongia mobilis*. The relative abundance of these species varied from 3.85 to 6.73%. In the case of sediment, the top 5 dominant species were *Sulfuricurvum kujiense, Holomonas phosphatis, Roseomonas lacus, Woodsholea maritima*, and *Desulfococcus biacutus* (Figure 5F). The relative abundance of genera varied from 5.4 to 10.8% (Figure 5E). The range of variation in relative abundance for sludge and sediment were 0.96–6.73% and 1.35–10.81%, respectively across the species (Supplementary Tables S1, S2).

Although the composition of microbial community at species level is different between sludge and sediment, but the abundance values are marginally different. In other words, the structure of microbial community at the identified species level is markedly different from that of the structure observed at higher taxonomic categories.

There are no abundant and common species in both the communities of sludge and sediment and almost all the species belong to very rare category except few of them that belong to rare category (Table 4). For example, in sediment 96% of species were very rare and very rare category was represented by 88.23%. Some of the top 5 dominant species belong to rare category and all others fall under very rare category (Table 4). Usharani (2019) provided a list of species but the list contained only unclassified and unidentified taxa and the only identified species was *Pseudomonas aeruginosa* which was also found in the sludge and sediment with relative abundance of 2.88% and reported to promote plant growth in stressed environment. Lam et al. (2015) reported *Nitrosomonas* from Municipal Waste Water treatment CW having temperature of 29.1°C–29.6°C. This species was observed in the sludge with relative abundance of 1.92%. Most of the species identified appear to be monotypic suggesting rich taxonomic diversity.

### Similarities and differences in microbial communities between sludge and sediment at OTU level

**Figure 6:**
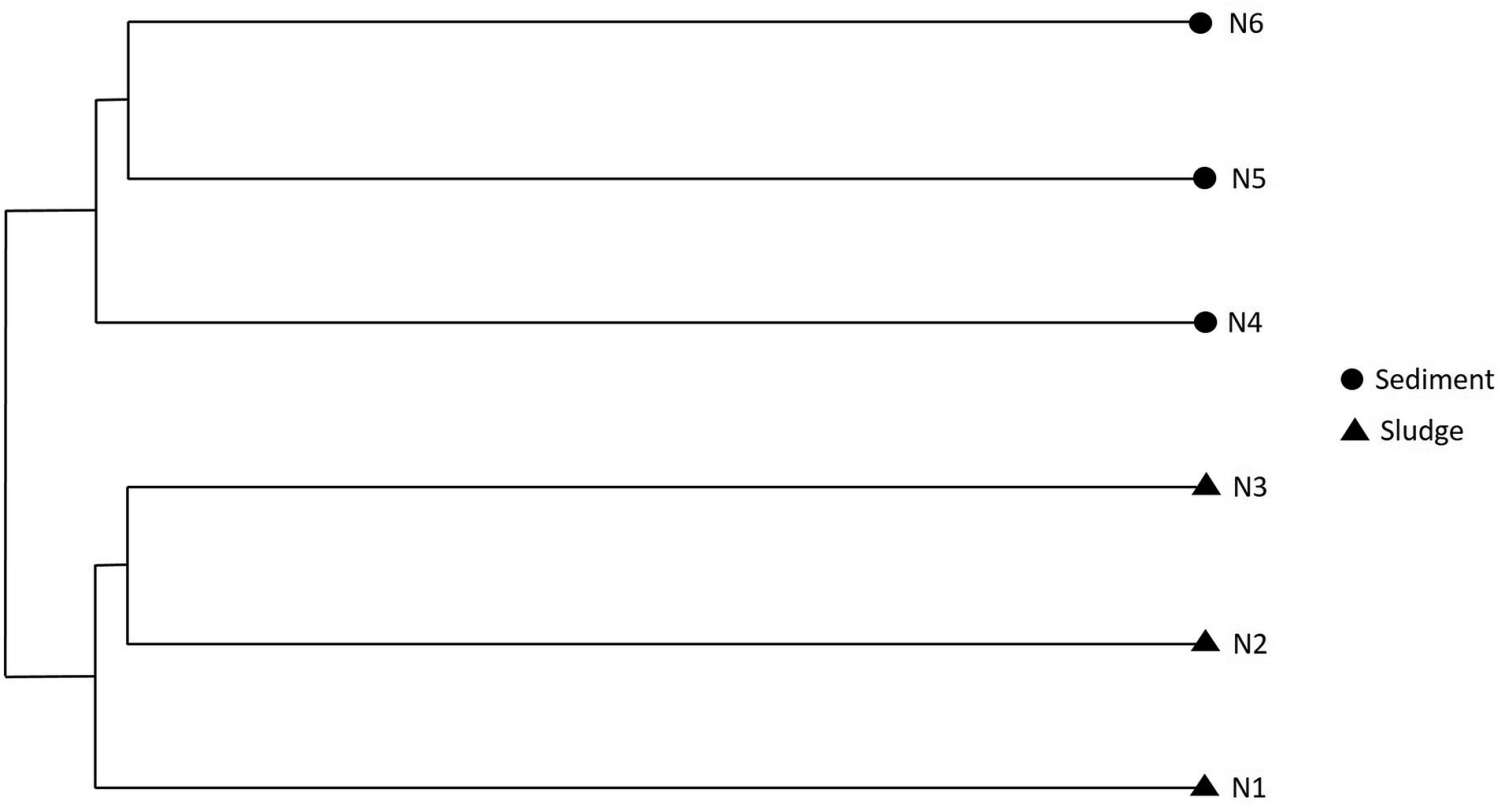
Phenogram showing the similarities and differences in the microbial communities (at OTU level) of samples of sludge and sediment.

Using Bray-Curtis distance coefficient, the OTUs were clustered. Two separate clusters corresponding to sludge and sediment were formed (Figure 6) suggesting the divergence in microbial communities between sludge and sediment. However, the samples within the sludge and sediment showed marked similarity.

## Conclusion

This is the first report of microbial community diversity in the integrated CWS used for *in situ* remediation of sewage and functional at Neela Hauz Biodiversity Park. The microbial community diversity in composition and structure are different quantitatively and qualitatively between sludge and sediment of the integrated CWS. These differences reflect different environmental factors and presence or absence of vegetation between stabilized pond from where sludge was sampled and CW from where sediment was collected. The combined microbiome of the sludge and sediment not only make the integrated CWS perform more efficiently in pollution removal than many of the CWs designed by other workers, but also make richer microbiome of CWS than that reported by other workers. This is essential as the microbial diversity present in the integrated CWS facilitates the biodegradation of toxicants and pollutants in the CWS. Characterization of microbial communities for the optimization of the pollutant removal can help in improving the efficiency of integrated wetland systems.

The unique features of the composition and structure of microbial communities of the integrated CWs are: (i) few taxa with high abundance values and several taxa with low abundance values at each taxonomic category ranging from phylum to species and (ii) the pattern of distribution of taxa among four categories of abundance showed a decrease in the number of abundant and common taxa and increase in rare and very rare taxa in descending order of categories (phylum to species) of the taxonomic hierarchy. Such structural organization of microbial community is perhaps associated with complex ecologies and environment stresses of the CWS studied.

Metagenomics of biofilms of rock filters in the filtration zone of the integrated CW may reveal novel microbial community and that will be different from what has been observed in the present study.

## Data availability statement

The datasets presented in this study can be found in online repositories. The names of the repository/repositories and accession number(s) can be found in the article/Supplementary material.

## Author contributions

NS: Conceptualization, Writing – original draft, Formal analysis, Investigation, Methodology, Software. SV: Supervision, Writing – review & editing. SL: Supervision, Writing – review & editing. YA: Writing – review & editing. SM-L: Writing – review & editing. MB: Writing – review & editing. CB: Conceptualization, Writing – original draft.
